# Optimization and Experimental Study of Iron Ore Grinding Medium Parameters Using EDEM Discrete Element Software

**DOI:** 10.3390/ma17194726

**Published:** 2024-09-26

**Authors:** Zhifeng Yin, Yuhang Zhang, Huajun Zhu, Hao Ding, Qisheng Wu, Zheyu Zhu, Jiming Song

**Affiliations:** 1 School of Materials Science and Engineering, Yancheng Institute of Technology, Yancheng 224051, China; zyuhang928@gmail.com (Y.Z.); qishengwu@ycit.cn (Q.W.); 17712937215@163.com (Z.Z.); jm68742@163.com (J.S.); 2 Anhui Provincial Key Laboratory of Green and Low-Carbon Technology in Cement Manufacturing, Hefei 230051, China; kinghao116@163.com; 3 Hefei Cement Research & Design Institute Corporation Ltd., Hefei 230051, China

**Keywords:** discrete element method, ball mill, particle breakage, energy saving

## Abstract

Energy savings and consumption reduction of ball mills are crucial for industrial production. The grinding medium is an important component of a ball mill. In theory, using higher-density grinding media can yield better grinding results. However, for materials with varying grindability, employing grinding media of different densities can reduce energy consumption while maintaining the same grinding effect. This study simulates the motion of the grinding media in the mill using three different densities of balls and the same material (iron ore). The results reveal that balls with densities of 5.8 g/cm^3^ and 7.8 g/cm^3^ achieve faster grinding of materials into finer particles, but balls with a density of 5.8 g/cm^3^ consume less energy. Therefore, replacing a ball with a density of 5.8 g/cm^3^ in a ball mill can significantly reduce energy consumption. This study will assist in selecting the optimal grinding medium density for different materials, ultimately contributing to energy savings and reduced carbon emissions.

## 1. Introduction

Ball mills are crucial equipment for grinding in the metallurgy, cement, chemical, and power industries. These mills contain cylinders filled with grinding medium balls that perform grinding operations on particles. Enhancing grinding efficiency, even marginally, can yield significant economic benefits. Given that ball mills typically operate with an efficiency below 15% and are highly energy intensive, they can account for up to 40% of the direct operating costs in a mineral processing plant [1,2]. Thus, selecting the optimal operating parameters is essential to improve grinding performance.

In the past, grinding media were considered integral to ball mills, and the effects of grinding ball size, the friction coefficient, and surface-to-volume ratio [3] were thoroughly investigated by Austin, L.G. et al. The influences of grinding medium material type and the friction coefficient [4,5] on milling performance were later studied by Umucu, Y. et al. In contemporary industrial production, one effective strategy to reduce the energy consumption of ball mills is to select alternative grinding medium materials, such as ceramic balls, to replace conventional steel balls [6]. The rationale for this substitution is that ceramic balls have a considerably lower density (approximately half that of steel balls) and a lower wear rate. According to the power consumption model [7], the power consumption of a ball mill is proportional to the density of the grinding balls. A positive relationship exists between the energy consumption of a ball mill and the density of the grinding balls. However, using alumina ceramic balls, which have only half the density of steel balls, can result in issues such as reduced yield and specific surface area (a measure used to characterize particle fineness). To address this, we propose utilizing a range of grinding media with varying density levels. For materials with differing grindability, employing grinding media of different densities can reduce energy consumption while achieving the same grinding effect. This approach reduces energy consumption while maintaining the desired yield and specific surface area.

With the advancement of computer power and optimization of algorithms, the discrete element method (DEM) has enabled effective simulation of the dynamic behavior of particles in a ball mill. This method treats the particle stream as a collection of individual particles and calculates their trajectories based on Newton’s second law. Within the discrete element system model, interactions between particles and between particles and the rotor are calculated according to internationally recognized principles of contact mechanics. Due to the high energy consumption of ball mill experiments, the discrete element method (DEM) has been found by scientists to simulate the operation of ball mills very well, greatly reducing the waste of energy generated by cumbersome experiments. Mishra, B.K. and Rajamani, R.K. were the first to apply a two-dimensional discrete element model (in 1992) to study and analyze the dynamics of the grinding media and material particles within the cylinder of a ball mill, as well as the impact of the grinding process on energy consumption [8]. As a result, this method has since been widely adopted in the study of various grinding processes. Since the ball mill is in a completely closed state when it is in operation, the trajectory of the ball milling media cannot be well observed, so scientists use the discrete element method to carry out simulations, which can easily observe the trajectory of the grinding media. Makokha, A.B. and Moys, M.H. from the University of Witwatersrand experimentally explored the motion laws of the grinding media in ball mills and analyzed them through numerical simulations using discrete element simulation software [9]. In 2011, in order to avoid the effect of lifting bar wear on ball milling results, Powell, M.S. et al. applied discrete element software EDEM 2006 to simulate the ball mill operation process, performed a predictive analysis of the wear rate of the lifting bar, and constructed a model to investigate the specific impact of the liner on ball mill ergonomics [10]. In order to reduce energy consumption, the researchers chose to change the grinding media material for grinding. Muanpaopong, N. and colleagues demonstrated the considerable effect of the ball material (e.g., steel and aluminum oxide) and its size on the fracture kinetics of pre-ground cement clinker in a laboratory-scale ball mill [11]. However, studies have only reported the use of alumina ceramic balls with a density of 3.8 g/cm^3^, and there are no academic reports on the influence of grinding medium materials with different densities on the correlation between grinding output and energy consumption. Overall, the study of ball mills using the discrete element method is designed to reduce energy consumption while maintaining grinding efficiency. Currently, only studies using grinding media with a density of 3.8 g/cm^3^ have been conducted, and no studies have been found on the use of different densities of grinding media for materials with different grindability. Therefore, the main focus of this study is to investigate the effects of different densities of grinding media using a combination of the discrete element method (DEM) and experimentation.

Despite considerable advances in computational power, transient simulations of dynamic systems using physical-numerical methods (e.g., DEM) remain costly for large-scale practical applications. Therefore, we propose the use of a laboratory mill as a prototype for simulation studies and for making industrial production predictions under various operating conditions to explore more possibilities regarding the use of different parameters.

The purpose of this study is to utilize EDEM 2022^®^ software simulations to investigate the impact of grinding media with different densities on the grinding effectiveness of iron ore. Moreover, the study proposes specific density grinding media tailored to specific materials to effectively reduce energy consumption while maintaining yield. That is, the creative point of this paper is to demonstrate that materials with different grindability need to be matched with grinding media of specific densities for grinding, which is more conducive to reducing energy consumption.

## 2. EDEM Model and Simulation Conditions

### 2.1. Model Description

The simulations were conducted using EDEM 2022^®^, a commercial software for high-performance DEM simulation. EDEM is the world’s first multi-purpose discrete element method (DEM) modeling software designed for simulating and analyzing production processes in industrial particle handling and related equipment. Its advantages include reducing the need for physical prototypes and experiments, providing insight into particle-scale behavior that is difficult to measure, and determining the effects of particle flow on fluids and machinery. EDEM offers various theories and methods for contact modeling, with the most commonly used models being Hertz–Mindlin (no slip), Hertz–Mindlin (no slip) with RVD (relative velocity-dependent) rolling friction, and Hertz–Mindlin (no slip) with JKR (Johnson Kendall Roberts) cohesion [12]. The Hertz–Mindlin (no slip) contact model is the default in EDEM owing to its efficient and accurate force calculations. In this model, the normal force component is based on Hertz contact theory, while the tangential force component follows the Mindlin–Deresiewicz model. Both normal and tangential forces include a damping component, and the damping coefficient is dependent on the coefficient of restitution. Tangential friction follows the Coulomb friction law. Rolling friction is implemented using a constant torque model independent of contact. Additionally, for granular material crushing, the Bonding V2 model is employed, allowing the bonding of smaller particles to form desired material particles by adjusting the bonding force [13].

The DEM model utilized in this study is based on the soft sphere model, which has been widely used to study a variety of extensively applied research phenomena such as particle accumulation, transport properties, hopper flow, mixing, and granulation. This model is particularly suitable for systems such as ball mills owing to its capability of managing multiple contacts concurrently. Particle motion is governed by Newton’s laws of motion. The equations governing the translational and rotational motion of particle *i* with mass *m_i_* and moment of inertia *I_i_* can be expressed as follows [14]:

(1) $$m_{i}\frac{dv_{i}}{dt}=\sum (F_{ij}^{n}+F_{ij}^{s}+m_{i}g)$$

And

(2) $$I_{i}\frac{d\omega_{i}}{dt}=\sum (R_{i}\times F_{ij}^{s}+M_{ij}^{r})$$

where *v_i_*, *ω_i_*, and *I_i_* represent the translational velocity, angular velocity, and moment of inertia of the particle, respectively; *R_i_* is the vector from the center of particle *i* to the contact point, and its magnitude is equal to the particle radius *R_i_*; and $F_{ij}^{n}$ and $F_{ij}^{s}$ denote the normal and tangential contact forces applied to particle *i* by particle *j*, respectively.

(3) $$F_{ij}^{n}=[\frac{2}{3}E\sqrt{\bar{R}}{\xi_{n}}^{\frac{3}{2}}-\gamma_{n}E\sqrt{\bar{R}}\sqrt{\xi_{n}}(v_{ij}\cdot n_{ij})]n_{ij}$$

And

(4) $$F_{\mathrm{ij}}^{s}=-\mathrm{sgn}(\xi_{s})\mu |F_{ij}^{n}|[1-{\left(1-\min(\xi_{s},\xi_{s,\max})/\xi_{s,\max}\right)}^{\frac{3}{2}}]$$

where $E=Y/(1-\tilde{\sigma^{2}})$; Y and $\tilde{\sigma }$ denote Young’s modulus and Poisson’s ratio, respectively; $\xi_{n}$ denotes the overlap between particles *i* and *j*; and *n_ij_* is the unit vector from the center of particle *j* to the center of particle *i*.
where $\bar{R}=R_{i}R_{j}/(R_{i}+R_{j})$; *γ_n_* is the normal damping constant; and $\xi_{s}$ and $\xi_{s,\max}$ are the total tangential displacement and the maximum tangential displacement of the particles during contact, respectively.

The Rayleigh time step is a critical factor in DEM simulation, representing the time required for a shear wave to travel through a solid particle. For DEM simulations of quasi-static particle collections, the theoretical maximum time step ensures that the coordination number (the total number of contacts per particle) remains above 1. The Rayleigh time step can be expressed as follows [13]:

(5) $$T_{R}=\frac{\pi R\sqrt{\frac{\rho }{G}}}{0.1631V+0.8766}$$

where *R* is the particle’s radius, *ρ* is its density, *G* is the shear modulus, and *V* is Poisson’s ratio.

### 2.2. Simulation Conditions

**Figure 1:**
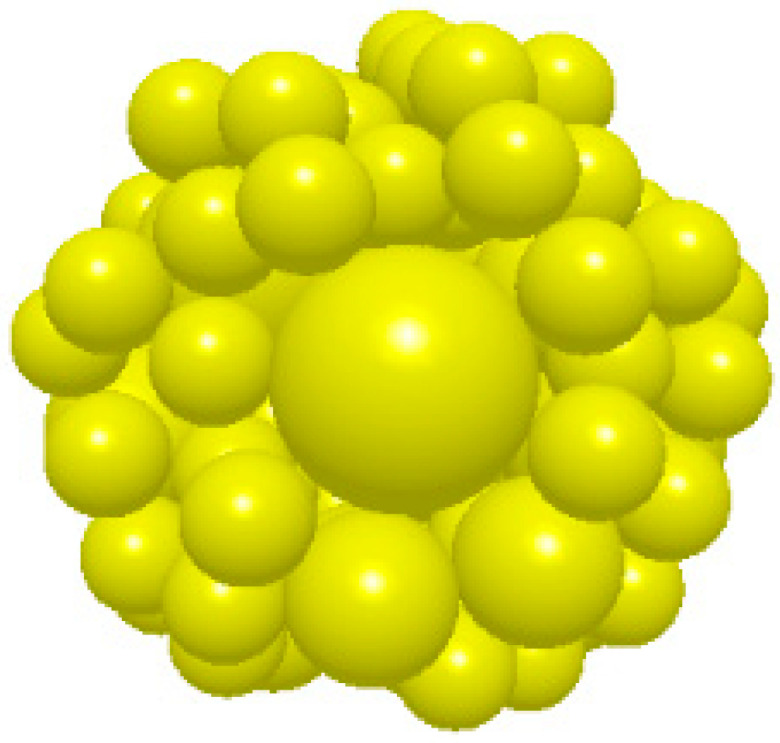
Shape of powder particles.

In the study, the adopted ball mill model had a diameter of 350 mm and a cylinder length of 350 mm. In this model, we designed 12 trapezoidal lifting bars, each 5 mm in height, uniformly distributed to achieve the desired lifting effect. A boundary 2 mm larger than the model’s dimensions was set to closely replicate the real model while preventing the spillage of material and grinding media. The number and diameter of the balls were determined based on the mill size, friction coefficient, and other parameters in conjunction with actual production practices. The grinding media consisted of 159 balls with a diameter of 20 mm, 57 balls with a diameter of 25 mm, 39 balls with a diameter of 30 mm, and 25 balls with a diameter of 40 mm. Iron ore powder particles with a diameter of <2.5 mm were used as the grinding material, and constituent particle diameters were randomly generated. The shape of the powder particles is illustrated in Figure 1, and the detailed simulation parameters are listed in Table 1. These parameters were mainly determined according to the actual measurement results and simulation attempts. Calculations were made based on the actual properties of these materials. The density of the grinding medium was varied in the simulation. During the simulation, variables such as the density, friction coefficient, Poisson’s ratio, and elastic modulus of the grinding medium were adjusted.

The simulation begins with the random generation of all particles with no overlap within the mill. These particles begin to fall under gravity and collide with each other until they settle into a stable position, forming a filled bed. Once the bed is established, the ball mill is rotated at a consistent speed. Once the ball mill operation reaches a steady state, data are collected and analyzed [15]. During the storage process, the calculated data may overflow and truncate, so it is necessary to go through several calculations and average each set of data to ensure the scientific accuracy of the data and avoid inaccuracies.

## 3. Results and Discussion

### 3.1. Effects of Grinding Medium Density on the Ball Motion Pattern and Flow Pattern

**Figure 2:**
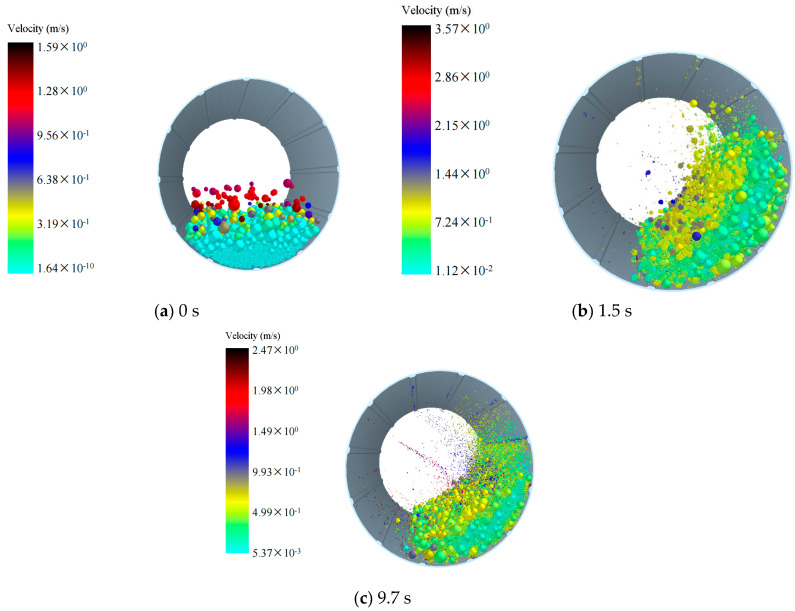
Motion patterns of the balls with a grinding medium density of 3.8 g/cm^3^: (**a**) 0 s; (**b**) 1.5 s; (**c**) 9.7 s.

**Figure 3:**
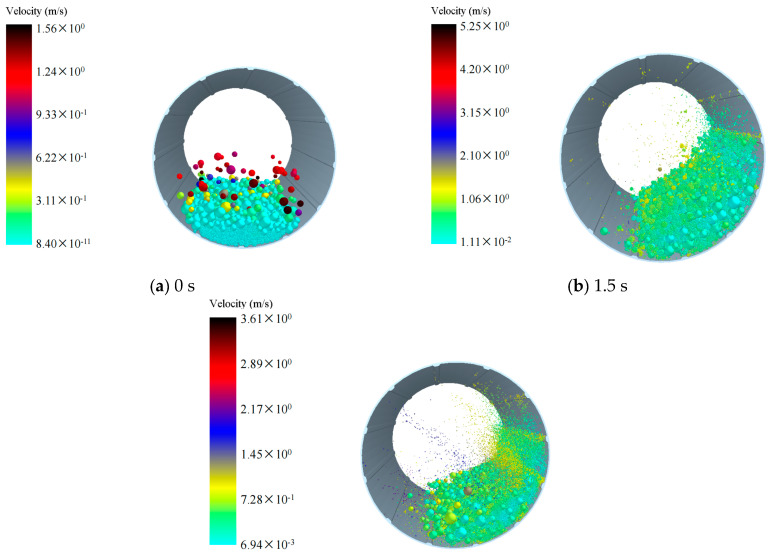
Motion patterns of the balls with a grinding medium density of 5.8 g/cm^3^: (**a**) 0 s; (**b**) 1.5 s; (**c**) 9.7 s.

**Figure 4:**
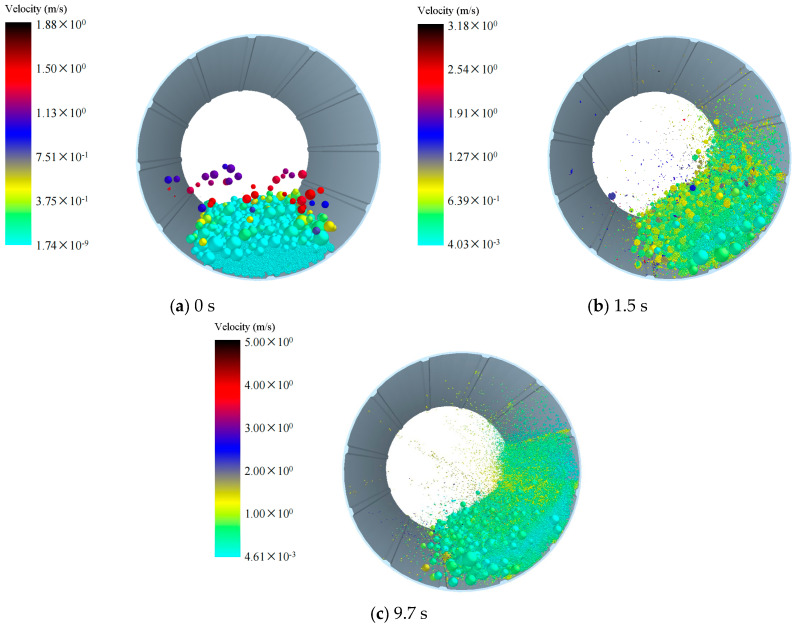
Motion patterns of the balls with a grinding medium density of 7.8 g/cm^3^: (**a**) 0 s; (**b**) 1.5 s; (**c**) 9.7 s.

We investigated the influence of different densities of grinding balls on ball motion patterns. Therefore, we simulated the ball motion patterns during mill operation, and Figure 2, Figure 3, Figure 4 illustrate the motion of grinding balls at varying densities. Under a grinding medium density of 3.8 g/cm^3^ (Figure 2), at 0 s, particles are just beginning to rotate after being generated, and the simulation starts with the formation of a filled bed of balls and material in a static mill (Figure 2a). At 1.5 s into the simulation (Figure 2b), the grinding balls and materials begin to make contact and grind together. By 9.7 s (Figure 2c), the majority of the materials have been ground and broken into small particles that are moving within the mill. Some larger, unbroken materials remain on one side of the mill, continuing to undergo grinding. Figure 3 and Figure 4 illustrate the ball movement patterns with grinding medium densities of 5.8 g/cm^3^ and 7.8 g/cm^3^, respectively. The ball movement patterns across the three grinding medium densities are largely similar. However, at 9.7 s, a considerably greater number of particles have been broken in the models with grinding medium densities of 5.8 g/cm^3^ and 7.8 g/cm^3^ than in the model with a grinding medium density of 3.8 g/cm^3^. These ball motion patterns are consistent with the results of previous studies [16,17,18].

As depicted in Figure 2, Figure 3, Figure 4, at 75 rpm, with the grinding balls filled to 30%, the flow patterns are similar, indicating that particles were lifted toward the shoulder of the mill. Across all cases, the heights of the flow shoulder are similar. However, with increasing grinding time, more smaller particles are crushed, and smaller, lighter balls are thrown toward the center of the mill. According to velocity graphs (Figure 2, Figure 3, Figure 4), particles in the center exhibit a notable increase in velocity, transitioning the flow pattern from cascade to waterfall. Moreover, larger particles and heavier grinding media remain on the flow shoulder, continuing in a cascade motion. This flow behavior aligns with observations reported in numerous studies [19,20].

### 3.2. Effects of Grinding Medium Density on Energy Distribution

Various aspects of ball mill energy have been extensively studied using EDEM. Previous studies have examined the impact energy [20,21]; collision energy [22]; and torque, power, and energy consumption of the mill [23]. Building on this body of research, this study investigated the grinding effects generated by grinding media with different densities, focusing on the collision energy, torque, power, and overall energy consumption of the ball mill.

In a ball mill, pellets experience a range of interactions with particles, grinding media, and the mill itself. The energy dissipated during these contacts is crucial for breaking down the particles, whereas energy expended on other interactions is considered wasted. Therefore, it is essential to assess how contacts involving grinding media of different densities contribute to the dissipation of energy between particles.

#### 3.2.1. Effects of Grinding Medium Density on Collision Energy

Energy dissipation from inter-particle interactions in a ball mill has been calculated using discrete element simulations. The analysis focuses on the frequency distribution of collision energy losses, encompassing tangential, normal, and overall energy losses. In EDEM software, collision energy is typically measured in joules (J). Understanding the distribution of this energy is crucial for researchers and practitioners in the fields of ore grinding and crushing, as energy distribution plays a crucial role in ore crushing and milling processes.

**Figure 5:**
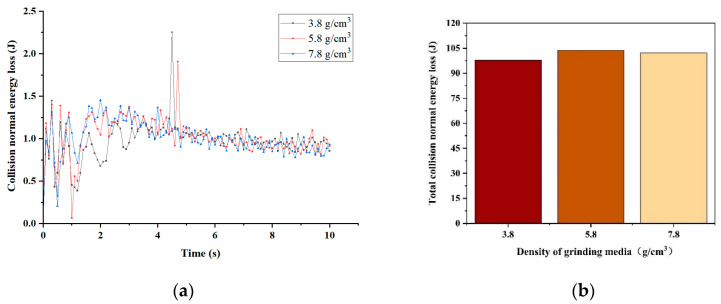
Normal energy loss: (**a**) collision normal energy loss; (**b**) total collision normal energy loss.

Therefore, we conducted simulations using grinding media with different densities. The results of the collision normal energy simulations for various grinding medium densities are presented in Figure 5. Figure 5a illustrates the collision normal energy generated between the grinding media and the material at each time point from 0 to 10 s. Most losses for all three types of grinding media fall within the range of 0.5–1.5 J. Figure 5b displays the total collision normal energy lost by each density of grinding media after 10 s of simulation. The collision normal energy obtained using grinding media with a density of 5.8 g/cm^3^ is considerably higher than that obtained using grinding media with a density of 3.8 g/cm^3^ and slightly greater than that obtained using grinding media with a density of 7.8 g/cm^3^. This illustrates that grinding media with a density of 5.8 g/cm^3^ undergo more alterations in instantaneous velocity during the simulation process, thereby achieving a relatively better grinding effectiveness.

**Figure 6:**
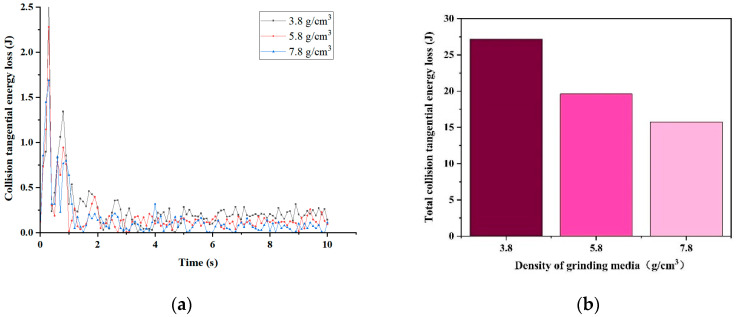
Tangential energy loss: (**a**) collision tangential energy loss; (**b**) total collision tangential energy loss.

Figure 6 displays the simulation results of collision tangential energy for grinding media with varying densities. In Figure 6a, the collision tangential energy generated between the grinding media and the material at each time point within 0–10 s is illustrated. Most losses for all three types of grinding media fall within the range of 0–0.5 J during stable model operation. Figure 6b displays the total collision tangential energy lost under each grinding medium density after 10 s of simulation. The total collision tangential energy lost decreases with increasing grinding medium density from 3.8 g/cm^3^ to 5.8 g/cm^3^ and 7.8 g/cm^3^. This trend is attributed to the lighter weight of the grinding media with a density of 3.8 g/cm^3^, which requires more energy loss to break the material during its curved motion. Consequently, the grinding effectiveness of grinding media with a density of 3.8 g/cm^3^ is considerably lower than that of the media with a density of 5.8 g/cm^3^ because the grinding media with a density of 3.8 g/cm^3^ is lighter, requiring more energy to break the material during curved motion. This indicates that the grinding effectiveness of the grinding media with a density of 3.8 g/cm^3^ is considerably lower than that of media with densities of 5.8 g/cm^3^ and 7.8 g/cm^3^.

**Figure 7:**
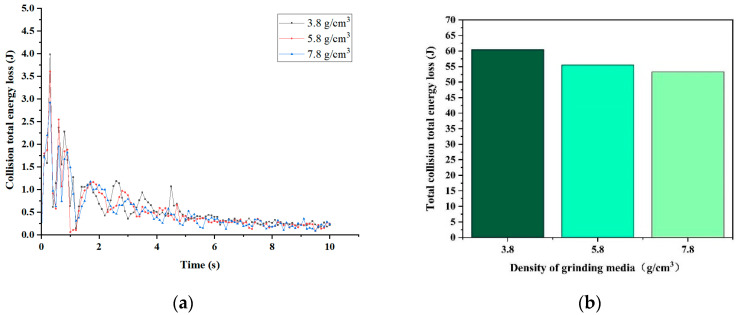
Collision energy loss: (**a**) collision total energy loss; (**b**) total collision total energy loss.

Figure 7 presents the overall collision energy loss for the three grinding medium densities. Figure 7a illustrates the energy loss at each moment, while Figure 7b summarizes the simulation results over 10 s. The total collision energy loss for each grinding medium density is illustrated in Figure 7b. The total collision energy lost decreases with increasing grinding medium density from 3.8 g/cm^3^ to 5.8 g/cm^3^ to 7.8 g/cm^3^. This trend suggests that grinding media with a density of 3.8 g/cm^3^ requires more collision energy during the grinding and crushing process to break the material. Therefore, the grinding effectiveness of media with a density of 3.8 g/cm^3^ is inferior to that of the other two types of grinding media.

#### 3.2.2. Effects of Grinding Medium Density on Particle Energy

Through the calculation of the potential energy generated by the grinding media and the material particles during the grinding process, the grinding effectiveness of the media on the granular material can be roughly assessed. Therefore, we installed detectors to measure potential energy and analyzed the energy generated using grinding media of varying densities.

**Figure 8:**
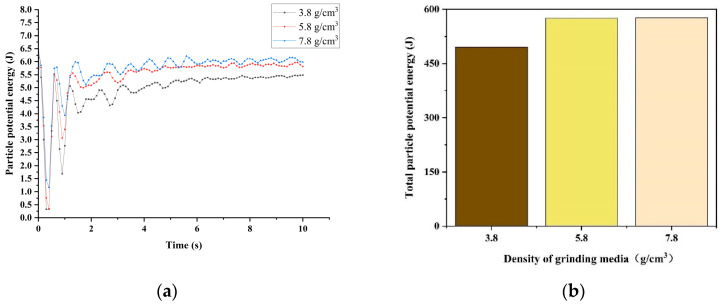
Potential energy: (**a**) particle potential energy; (**b**) total particle potential energy.

As illustrated in Figure 8, the potential energy generated by material particles during the milling process varies with the milling medium density. Greater potential energy generally results in a more effective impact and better crushing and grinding effects. As depicted in Figure 8a, at each moment, the grinding media with a density of 7.8 g/cm^3^ correspond to the largest potential energy, followed by the grinding media with a density of 5.8 g/cm^3^, and then the media with a density of 3.8 g/cm^3^. Figure 8b illustrates the overall potential energy accumulated by the entire ball mill over 10 s under the three grinding medium densities. The potential energy associated with the grinding medium density of 3.8 g/cm^3^ is lower than that corresponding to densities of 5.8 g/cm^3^ and 7.8 g/cm^3^, whereas the potential energy obtained by the model of the ball mill with a grinding medium density of 5.8 g/cm^3^ is rather close to that of the ball mill model with a grinding medium density of 7.8 g/cm^3^. Thus, the grinding media with a density of 5.8 g/cm^3^ also provide superior grinding effectiveness.

### 3.3. Influence of the Grinding Effectiveness

In this study, the material particles were bonded together to form the desired material structure. Therefore, the number of bond breaks was utilized in this paper to accurately measure grinding effectiveness during the simulations. We set up a detector to detect bond breakage and assessed the bond breakage resulting from the grinding of iron ore particles under three grinding medium densities.

**Figure 9:**
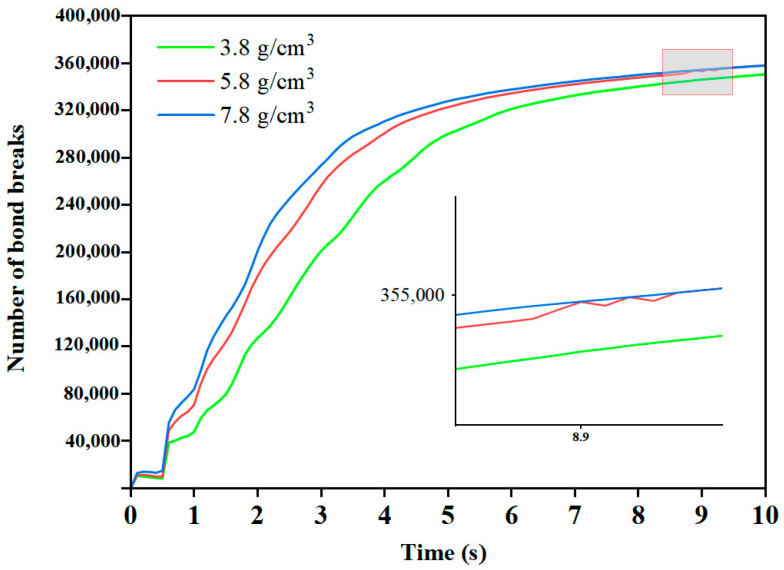
Number of bond breaks.

The grinding effects achieved by the three grinding medium densities over 10 s are depicted in Figure 9. Once the mill stabilizes, the number of bond breaks is consistently highest in the mill with a grinding medium density of 7.8 g/cm^3^ and lowest in the mill with a grinding medium density of 3.8 g/cm^3^. Around 8.9 s, the number of bond breaks in the mill with a grinding medium density of 5.8 g/cm^3^ equals or exceeds that in the mill with a grinding medium density of 7.8 g/cm^3^. This observation indicates that the grinding media with a grinding medium density of 5.8 g/cm^3^ provide superior grinding effectiveness, gradually reducing the particle size of the granular material.

**Figure 10:**
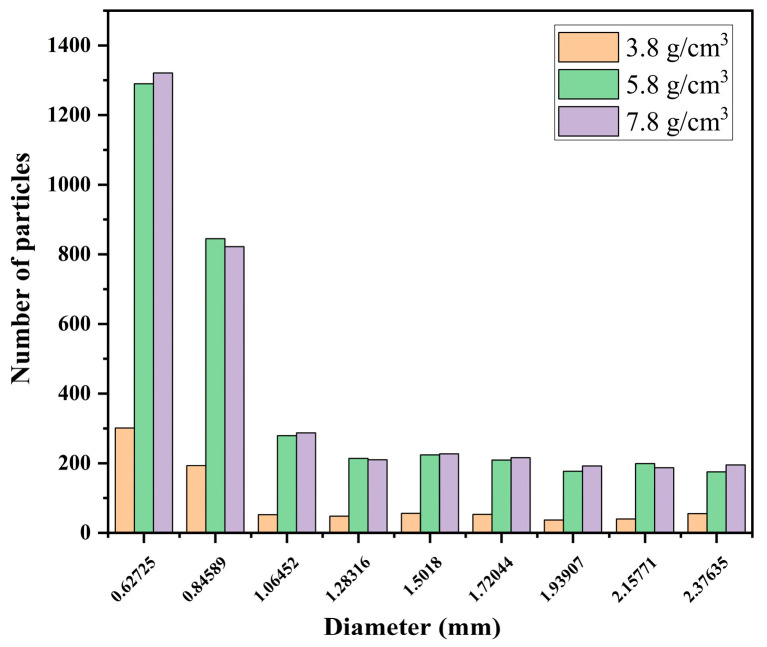
Particle size distribution at 9 s.

According to the simulations described above, we developed an auxiliary validation model that was verified against the Tavares model, a widely used crushing model in EDEM [23]. The particle size distribution of the material by the 9th second of simulation under the same simulation conditions as presented above is illustrated in Figure 10. At a small particle size, the grinding medium density of 5.8 g/cm^3^ results in a comparable or slightly better grinding effectiveness than the grinding medium density of 7.8 g/cm^3^.

### 3.4. Influence of Grinding Medium Density on Torque, Power, and Energy Consumption

In practical production processes, the torque, power, and energy consumption of the mill are important factors, and reducing energy consumption while increasing yield and specific surface area is a vital objective [24,25,26]. Therefore, we conducted discrete element simulations to analyze the torque, power, and energy consumption of the mill under three grinding medium densities.

**Figure 11:**
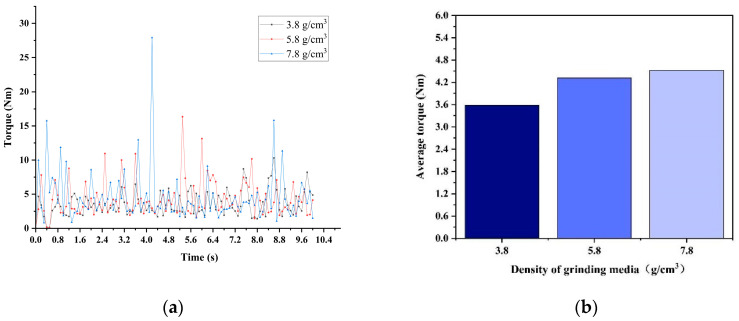
Torque: (**a**) instantaneous torque; (**b**) average torque.

Figure 11 presents the torque data obtained from simulations using EDEM discrete element software, in which different grinding medium densities are employed. The high accuracy of the software ensures the clarity of the captured mill torque. The instantaneous torque at each moment is accurately depicted in Figure 11a. Snapshots of the instantaneous torque at different moments revealed considerable fluctuations in the ball mill torque. The EDEM software is highly responsive to changes in these instantaneous torque values. To quantify the ball mill torque values (Figure 11b), precise measurements are necessary. Figure 11b illustrates the average torque of the three grinding medium densities within the mill over a 10 s period. The average torque of the ball mill increases with the increase in grinding medium density from 3.8 g/cm^3^, to 5.8 g/cm^3^, and 7.8 g/cm^3^. The average torque values under densities of 5.8 g/cm^3^ and 7.8 g/cm^3^ are relatively close. Torque directly influences the power of the mill, which is calculated as the product of torque and rotational speed.

**Figure 12:**
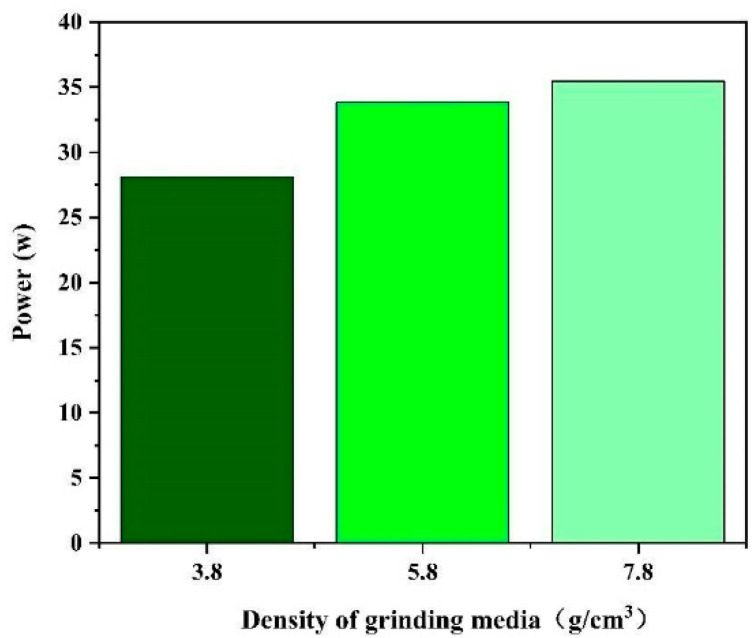
Power in 10 s.

**Figure 13:**
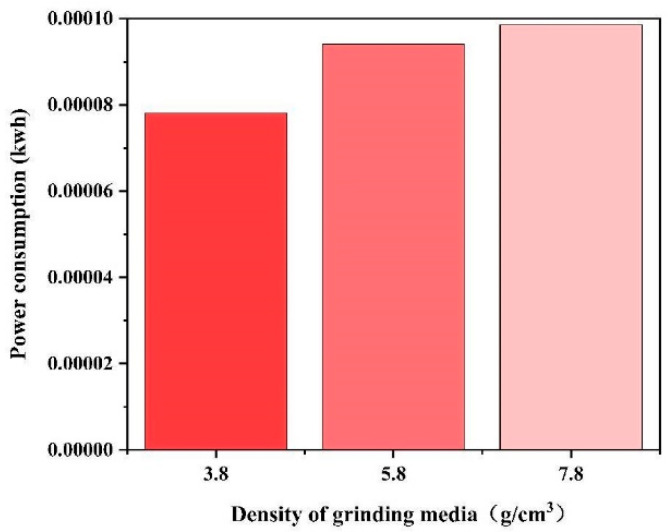
Power consumption within 10 s.

We studied the power consumption of the mill under the three grinding medium densities. The power consumption at the locations of the three different grinding media can be observed in Figure 12. Considering that the mill speeds are all constant at 75 rpm, the power trend is the same as that of the torque. Energy consumption, often referred to as the integral of power over time, is determined through the multiplication of power with time. This relationship allowed us to calculate the energy consumed during grinding with three different grinding media over 10 s (Figure 13).

The calculations reveal that the use of a grinding medium density of 3.8 g/cm^3^ saves 20.7% in energy consumption compared with the use of a density of 7.8 g/cm^3^. Similarly, the use of a grinding medium density of 5.8 g/cm^3^ saves 4.6% in energy consumption compared with the use of a density of 7.8 g/cm^3^.

### 3.5. Validation of Experimental Results

According to the EDEM simulations, we constructed a laboratory ball mill with the same dimensions as the model, 350 mm in diameter and 350 mm in cylinder length, and we tested it using two grinding media densities. From the simulation results, we selected two grinding media with optimal density effects: 5.8 g/cm^3^ and 7.8 g/cm^3^. The grinding material used was iron ore particles with a size of less than 2.5 mm, and the mill speed was set to 75 rpm. We tested the two grinding media for screen and specific surface area values at 10 min intervals from 60 to 150 min, as well as for power consumption during grinding.

**Figure 14:**
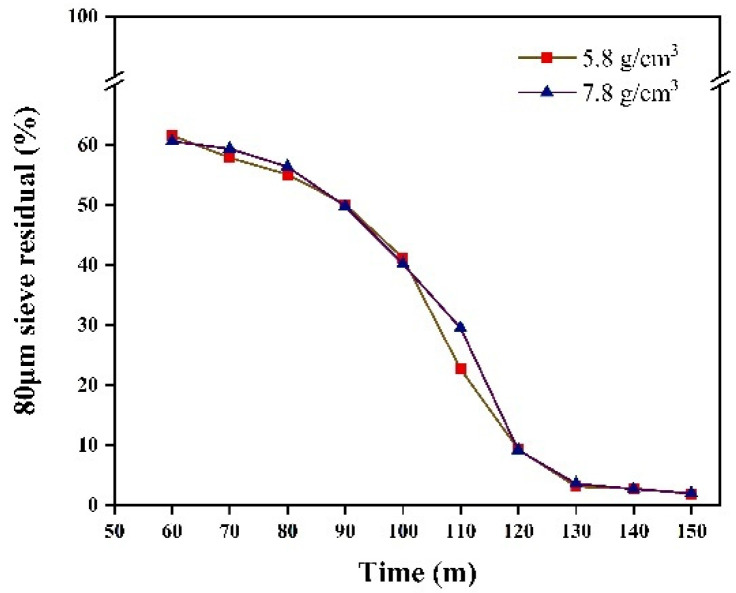
80 μm sieve residual.

**Figure 15:**
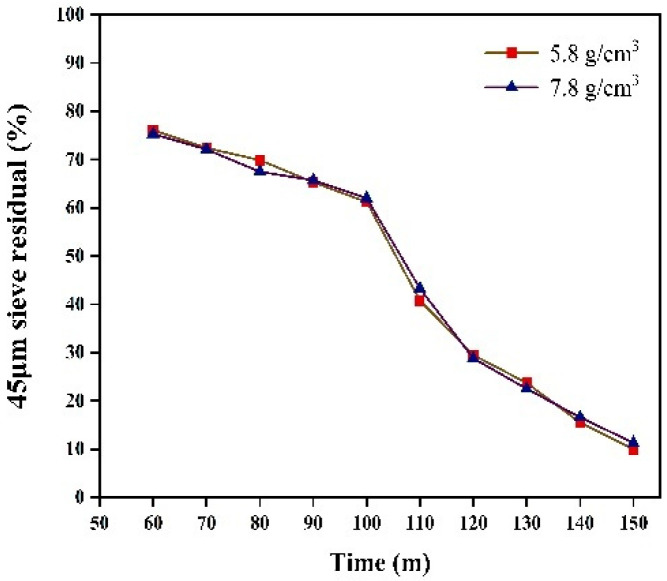
45 μm sieve residual.

**Figure 16:**
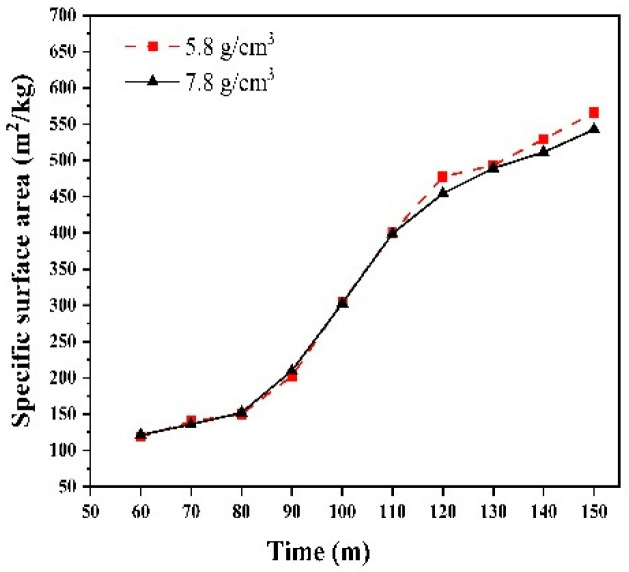
Specific surface area.

We investigated the sieve residue under the two different grinding medium densities. Sieve residue tests were conducted using a negative pressure sieve analyzer, in accordance with the Chinese industry standard JTG 3432-2024 [27]. Figure 14 shows the 80 μm sieve residues collected at 10 min intervals from 60 to 150 min for grinding medium densities of 5.8 g/cm^3^ and 7.8 g/cm^3^. The grinding effects were similar for both densities, with comparable fineness achieved at the same grinding time. Additionally, the 45 μm sieve residues under the two densities were also similar (Figure 15). Additionally, in industrial production, the use of ceramic balls to reduce energy consumption may lead to a decrease in the specific surface area. Therefore, we investigated the effect of different grinding medium densities on the specific surface area after grinding. Specific surface area tests were conducted using a specific surface area meter, in accordance with the Chinese national standard GB/T 8074-2008 [28]. Figure 16 illustrates the specific surface area of the materials after grinding under two grinding medium densities. The grinding effects of the two grinding media are nearly identical before 120 min. However, from 120 to 150 min, the grinding medium density of 5.8 g/cm^3^ results in a slightly better grinding effect than the density of 7.8 g/cm^3^. When the grinding media with a density of 5.8 g/cm^3^ are used to grind iron ore particles smaller than 2.5 mm, the specific surface area achieved is comparable to that obtained with the grinding media with a density of 7.8 g/cm^3^.

**Figure 17:**
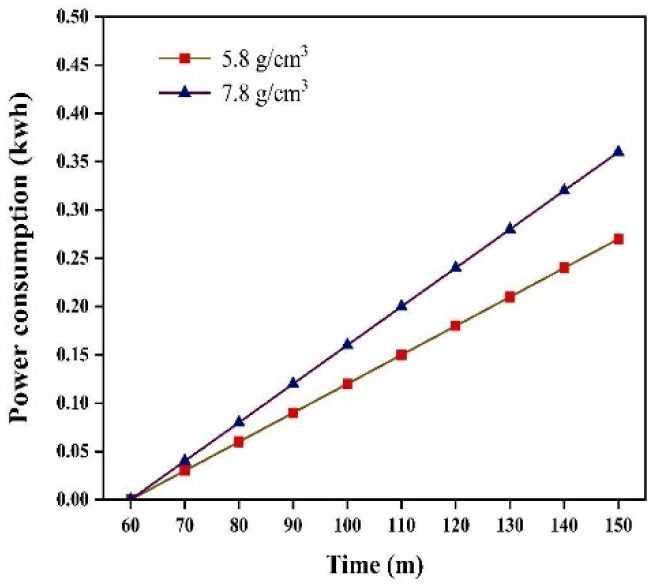
Power consumption.

Figure 17 shows the power consumption recorded at 10 min intervals from 60 to 150 min for grinding medium densities of 5.8 g/cm^3^ and 7.8 g/cm^3^. Using the grinding media with a density of 5.8 g/cm^3^ resulted in a 25% reduction in energy consumption compared to that obtained using the griding media with a density of 7.8 g/cm^3^.

## 4. Conclusions

For materials with varying grindability, using grinding media of different densities can reduce energy consumption while achieving the same grinding effect. This study investigated the effect of grinding medium density (3.8 g/cm^3^, 5.8 g/cm^3^, and 7.8 g/cm^3^) on the grinding of iron ore in a laboratory scale ball mill, focusing on energy consumption analysis. A ball mill model was developed according to DEM simulation data to evaluate grinding efficiency and energy consumption. Collision energy, bond breakage count, and particle size data obtained from DEM simulations were employed to characterize the grinding efficiency, while torque and power measurements were employed to assess energy consumption. The accuracy of the simulations and the reliability of the results were verified through experimental measurements of the material parameters and calibration of the model, as well as through multiple simulations and experimental verification. The key findings of the study are summarized as follows:

1. According to the EDEM simulations of the iron ore grinding process with particles smaller than 2.5 mm, the grinding efficiencies under grinding medium densities of 7.8 g/cm^3^ and 5.8 g/cm^3^ were comparable. However, the grinding medium density of 5.8 g/cm^3^ was more suitable, as it could theoretically result in considerable energy savings.
2. According to the comprehensive crushing kinetics analysis facilitated by DEM, we further evaluated the energy savings potential under grinding medium densities of 7.8 g/cm^3^, 3.8 g/cm^3^, and 5.8 g/cm^3^ in ball mills. Our findings indicated the following: (i) The use of the grinding medium density of 3.8 g/cm^3^ reduced energy consumption by approximately 20.7% compared with the use of the grinding medium density of 7.8 g/cm^3^. However, the grinding efficiency under the density of 3.8 g/cm^3^ was considerably lower than that under the density of 7.8 g/cm^3^. (ii) The use of the grinding medium density of 5.8 g/cm^3^ resulted in ~4.6% lower energy consumption compared with the use of the grinding medium density of 7.8 g/cm^3^. The grinding efficiency of iron ore under the grinding medium density of 5.8 g/cm^3^ was comparable to that under the density of 7.8 g/cm^3^. Moreover, the grinding efficiency under the grinding medium density of 5.8 g/cm^3^ surpassed that under the grinding medium density of 7.8 g/cm^3^ when the particle size was decreased.
3. The simulation accuracy was validated using operational data from a laboratory-scale mill. The findings of this study provide practical guidance for industrial production to reduce energy consumption and enhance energy utilization efficiency. However, the effects of large ball mills used in actual production and the grinding performance with materials other than iron ore remain unknown. Future studies should verify these results in larger ball mills and explore various materials to determine the optimal grinding density for different types of material.

## Figures and Tables

**Table 1:** Key parameters in the simulations.

Parameter	Base Value (Varying Values)
Mill length, L (mm)	350
Mill diameter, D (mm)	350
Rotation speed, Ω (rpm)	75
Ball density, ρ_b_ (kg/m^3^)	7800 (6000, 3800)
Particle density, ρ_p_ (kg/m^3^)	4372
Ball size, d_b_ (mm)	20, 25, 30, 40
Particle size, d (mm)	<2.5
Ball Young’s modulus, Y_b_ (GPa)	200 (150, 341)
Particle Young’s modulus, Y_p_ (GPa)	2.5
Ball Poisson’s ratio, σ	0.22 (0.2, 0.3)
Particle Poisson’s ratio, σ	0.25
Ball-to-ball collision recovery coefficient	0.9 (0.8, 0.5)
Ball-to-particle collision recovery coefficient	0.7 (0.6, 0.5)
Cylinder wall-to-ball collision recovery coefficient	0.7 (0.5, 0.3)
Cylinder wall-to-particle collision recovery coefficient	0.7 (0.6, 0.5)
Particle-to-particle collision recovery coefficient	0.5
Ball-to-ball static friction coefficient	0.4 (0.6, 0.8)
Ball-to-particle static friction coefficient	0.5 (0.4, 0.3)
Cylinder wall-to-ball static friction coefficient	0.6 (0.5, 0.4)
Cylinder wall-to-particle Static friction coefficient	0.7 (0.6, 0.5)
Particle-to-particle static friction coefficient	0.2
Ball-to-ball rolling friction coefficient	0.06 (0.08, 0.04)
Ball-to-particle rolling friction coefficient	0.07 (0.06, 0.05)
Cylinder wall-to-ball rolling friction coefficient	0.06 (0.05, 0.04)
Cylinder wall-to-particle rolling friction coefficient	0.04 (0.03, 0.02)
Particle-to-particle rolling friction coefficient	0.05
Normal stiffness, N/m^2^	100,000,000
Tangential stiffness, N/m^2^	500,000,000
Critical normal stress, Pa	500,000
Critical tangential stress, Pa	250,000

## Data Availability

The original contributions presented in the study are included in the article, further inquiries can be directed to the corresponding author.
